# Anion–Anion Interactions in Aerogen-Bonded Complexes. Influence of Solvent Environment

**DOI:** 10.3390/molecules26082116

**Published:** 2021-04-07

**Authors:** Anna Grabarz, Mariusz Michalczyk, Wiktor Zierkiewicz, Steve Scheiner

**Affiliations:** 1 Faculty of Chemistry, Wrocław University of Science and Technology, Wybrzeże Wyspiańskiego 27, 50-370 Wrocław, Poland; anna.grabarz@pwr.edu.pl (A.G.); mariusz.michalczyk@pwr.edu.pl (M.M.); 2 Department of Chemistry and Biochemistry, Utah State University, Logan, UT 84322-0300, USA

**Keywords:** molecular electrostatic potential, π-hole, AIM, energy decomposition

## Abstract

Ab initio calculations are applied to the question as to whether a AeX_5_^−^ anion (Ae = Kr, Xe) can engage in a stable complex with another anion: F^−^, Cl^−^, or CN^−^. The latter approaches the central Ae atom from above the molecular plane, along its C_5_ axis. While the electrostatic repulsion between the two anions prevents their association in the gas phase, immersion of the system in a polar medium allows dimerization to proceed. The aerogen bond is a weak one, with binding energies less than 2 kcal/mol, even in highly polar aqueous solvent. The complexes are metastable in the less polar solvents THF and DMF, with dissociation opposed by a small energy barrier.

## 1. Introduction

Interactions through σ- and π-holes are responsible for the formation of a wide group of noncovalently bound complexes [1,2,3,4,5]. Both of these sorts of hole originate in the anisotropic distribution of electron density, as for example in a depletion arising along extensions of covalent bonds to electron-withdrawing substituents, or in regions lying above a molecular plane. Depending upon the identity of the atom which has acquired such a positive region, the ensuing bonds are typically labeled as halogen [6,7,8,9], chalcogen [10,11,12], pnicogen [13,14,15,16], tetrel [17,18,19,20], triel [21,22,23], or aerogen bonds [24,25,26,27]. The underlying nature of these interactions have been the subject of considerable theoretical research, and have applications in fields such as crystal engineering [28,29,30,31,32,33], supramolecular chemistry [34,35,36,37], materials chemistry [38,39], and biochemistry [40,41,42,43]. A number of experimental works have generated an impressive database of crystalline structures which inspire detailed theoretical analyses. These same ideas have been extended to the nominally unreactive inert gas atoms, which Bauza and Frontera [27] dubbed the aerogen bond. While the aforementioned noncovalent bonds are generally associated with atoms commonly found on earth, often important components of biological structures, or participating in chemical reactions, the noble gases are characterized by their rare occurrence and low reactivity, so their participation in these noncovalent bonds was not entirely expected.

There has not been a great deal of past study of the aerogen bond (AeB). Most previous works have been devoted to complexes of noble gas oxides [44,45,46,47,48,49]. For example, Miao et al. examined geometries and spectral properties of several small molecular clusters containing XeO_3_ [47]. A series of DFT computation found binding energies of the more stable conformations of dimers are larger than in excess of 10 kcal/mol, and twice that for trimers. Another study [48] combined XeO_3_ with benzene, again yielding complexation energies on the order of 10 kcal/mol, either with various DFT functionals or with CCSD(T)/CBS. The binding is considerably weaker, however, less than 3 kcal/mol, for heterocyclic derivatives of benzene [50]. Our own group has previously considered AeBs between AeOF_2_ and diazines. AeOF_2_ contained both σ and π-holes; the former engaged in AeBs of up to 18 kcal/mol, as compared to the weaker bonds of the π-holes in the 6–8 kcal/mol range [46]. Similar energies were obtained by Gomila and Frontera for various complexes which appeared in the ICSD database [51] of xenon fluorides with a number of electron donors. Like their related noncovalent bond counterparts, AeBs are also subject to cooperative effects [45,49]. Chain elongation of the (KrOF_2_)_n=2–6_ and (XeOF_2_)_n=2–6_ clusters strengthened the individual bonds, more for the latter than for the former [45]. Likewise, the presence of an AeB strengthens a neighboring halogen bond [49]. With respect to individual Ae atoms, Carvalho and co-workers [52] provided an experimental benchmark to their computations of Ae···methanol complexes in the gas phase, finding that the binding strengthened along with increasing Ae atom size, from −0.4 to −3.9 kJ/mol. Their energy decomposition documented the importance of dispersion to this bonding. Similar conclusions were drawn by de Araujo Oliviera et al., for complexes between H_2_S and noble gases [53].

The forgoing papers, along with others, suggest that aerogen bonding follows the same patterns as the more extensively studied pnicogen or halogen bonds. However, surprisingly little is known about the impact of solvent on noncovalent bonds. In 2011 Lu et al., compared the interaction energies of iodo-perfluoroalkenes and -arenes, with halide ions, ammonia, and water in the gas phase and three different solvents [54]. Their results indicated that the bond strengths significantly weaken in solution, and is accompanied by elongation of the intermolecular distances. For example, the interaction energy of C_2_F_3_I···Cl^−^ in the gas phase is −26.2 kcal/mol, while placing this complex in chloroform results in almost a four-fold drop. In another set of systems, solvent caused a slight shortening of halogen bonds in neutral systems and relatively small changes in their energetics [54]. On the other hand, Bania et al. found that the change from vacuum to polar solvent reverses the negative interaction energies of cation–π complexes formed between light metal cations and substituted benzenes and borazines to positive values [55]. There has also been some study of the effects of solvents on cooperativity [25,56,57]. Esrafili’s group described the tuning of pnicogen and chalcogen bonds by aerogen-bonding in the presence of solvent [57], finding that the immersion in solvent reduces the interaction energies of binary and ternary complexes. Additionally, the increase of the solvent’s dielectric constant elongated the Ae···N distances, indicative of a weaker bond. These results are consistent with the weakening of pnicogen and chalcogen bonds in the presence of solvent [25].

While a primary effect of immersion in solvent appears to be a general weakening of the pertinent noncovalent bond, there have been a number of recent reports of a more drastic change when the two species involved are ions of like charge. Despite the Coulombic repulsions that keep these ions apart in the gas phase, charge dispersal effects accompanying solvation can allow them to approach close enough together so as to overcome the electrostatic repulsion and engage in a stable complex. After initial findings of this effect in the case of H-bonds [58,59,60,61,62,63,64,65,66,67,68,69], more recent work has shown these ideas can be extended to halogen [70,71,72,73,74,75,76,77], triel [78], pnicogen [79], and related types of noncovalent bonds [80,81,82,83,84]. There is an important question as to whether aerogen bonds, which are generally much weaker than most of the other related interactions, can likewise occur between pairs of anions, and if so, how strongly polarizing a solvent is needed.

The present work attempts to address this question via quantum chemical calculations. Potential AeB donors place Kr and Xe within the context of a AeX_5_^−^ anion where X refers to either F or Cl. The planar D_5h_ geometry of these anions has the potential to induce a relatively positive π-hole directly above the Ae atom which might attract a nucleophile. Anionic nucleophiles chosen to interact in this way are F^−^, Cl^−^, and CN^−^, all of which are compact so avoid dispersal of their charge over an extended system, and to avoid secondary interactions which might blur the results. In order to directly assess the effect of the solvent in a measured manner, three different solvents were chosen. Tetrahydrofuran (THF) is the least polar with a dielectric constant ε = 7.4. Dimethylformamide (DMF) is considerably more polar, with ε = 37.2, and water is strongest in this regard with a dielectric constant of 78.4. The possibility of each of the Ae-containing Lewis acids binding to each of the three anions is considered in each of these solvents, monitoring the strength of any bonding in each case. An inspiration for the choice of model system is derived from an important X-ray structure [84] of the pentafluoroxenate(IV) anion (XeF_5_^−^) [85] which represents the first reported example of a pentagonal planar specimen including an aerogen atom.

## 2. Computational Methods

Geometries of all monomers and their complexes were optimized at the MP2/aug-cc-pVDZ level [86,87] of theory. The pseudopotential aug-cc-pVDZ-PP basis was used for Xe atoms in order to incorporate relativistic effects [88]. This basis set has proven its accuracy and reliability for systems of this type in numerous comparisons with larger basis sets and with various levels of treatment of electron correlation [89,90,91,92,93,94,95,96,97,98,99]. To take into account solvent effects (solvents tetrahydrofuran THF, water, and *N*,*N*-dimethylformamide DMF), calculations utilized the Polarizable Continuum Model (PCM) in its linear response (LR-PCM) variant [100]. Harmonic frequency analysis verified that all optimized structures were in fact true local minima, with no imaginary frequencies. In the next step the interaction energy (E_int_) and the binding energy (E_b_) were calculated as the difference in energy between the complex and the sum of the two monomers. E_int_ placed the constituent monomers in their geometry within the complex, whereas E_b_ takes as its reference the monomers in their fully optimized isolated geometries. These two quantities thus differ by the deformation energy E_def_ induced by the complexation process on the geometries of the two subunits. Both quantities were corrected for the basis set superposition error (BSSE) via the counterpoise protocol defined by Boys and Bernardi [101].

Calculations were carried out within the framework of the latest version of the Gaussian 16 (C.01) program package [102]. QTAIM methodology was used to identify bond paths and their quantitative features through analysis of the electron density topology embedded in the AIMAll program [103]. The decomposition of the interaction energies was carried out by the LMO-EDA method based on the original Kitaura and Morokuma scheme [104] at the M06-2X/aug-cc-pVDZ level using MP2 optimized geometries (implemented in the GAMESS-US 2014 software) [105]. In this method, the total interaction energy is decomposed into electrostatic, exchange, repulsion, polarization and dispersion components [106]. The molecular electrostatic potential (MEP) and its extrema on the 0.001 au electronic isodensity surface, or at other particular points, were evaluated via the MultiWFN [107,108] and visualized by VMD [109] programs.

## 3. Results

### 3.1. Monomers

The AeX_5_^−^ anions are planar with approximate D_5h_ symmetry. The enlargement of the Kr atom to Xe elongates the Ae-F bond by some 0.03 Å, and a much larger increment of 0.66 Å occurs when F is replaced by Cl. There is very little effect of the nature of solvent or its dielectric constant on the internal geometry. It might be noted that KrCl_5_^−^ does not yield a stable structure, likely due to steric factors. The smaller size of Kr with its covalent radius of 1.16 Å, relative to Xe with r_cov_ = 1.40 Å, may not allow the five Cl atoms to space out sufficiently. This effect is more important for the larger Cl as compared to F. The computed bond lengths of XeF_5_^−^ can be compared with earlier works. The 2.012 Å average Xe-F distance found empirically by Christe and co-workers [85] is somewhat shorter than in our calculation. Grant et al. [110], on the other hand, observed a length of 2.034 Å in the gas phase, right along the lines of our own quantities.

**Figure 1:**
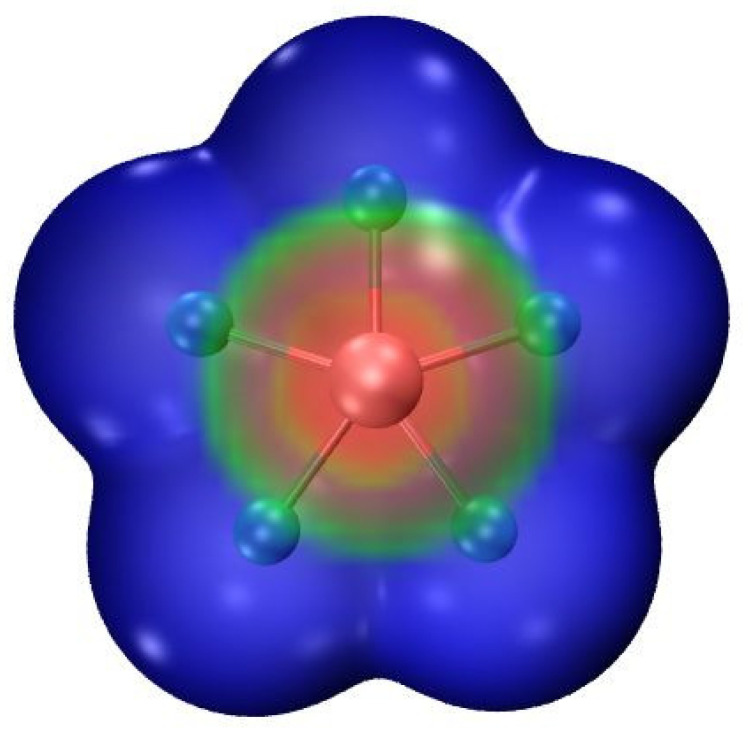
MEP of XeF_5_^−^ on its 0.001 au isodensity surface at the MP2/aug-cc-pVDZ level. Color scale ranges from −0.13 (blue) to −0.11 (red) au.

The molecular electrostatic potential (MEP) of XeF_5_^−^ is presented in Figure 1 and is emblematic of all three such anions. The potential is of course negative throughout as this entity is an anion. Its least negative region lies directly above and below the central Ae atom in what may be thought of as a π-hole. As may be seen in the second column of Table 1, the maximum of the MEP which is designated as V_S,max_ is most negative for KrF_5_^−^. It becomes somewhat less negative for the larger central Xe atom, and then takes a larger jump if the five F atoms are all replaced by the less electronegative Cl. Scanning down further in Table 1 makes it clear that the polarity of the solvent has little influence upon the MEP.

### 3.2. Complexes

**Figure 2:**
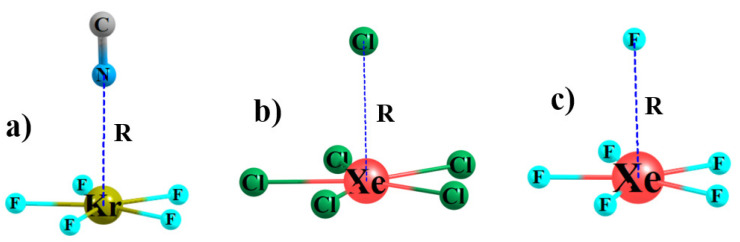
Optimized geometries of sample anion-anion complexes (**a**) KrF_5_^−^···NC^−^, (**b**) XeCl_5_^−^···Cl^−^, and (**c**) XeF_5_^−^···F^−^.

In keeping with the topology of the MEP of each AeX_5_^−^ anion, another anion would favor approach from directly above the central Ae atom, as illustrated in Figure 2 for some sample complexes. The diatomic CN^−^ can interact with the central Ae atom through either its C or N atom (see Table S1), but calculations found it is the interaction via N that was preferred, so it is this orientation which is described below. The intermolecular distances between the central Ae and the anion are listed in Table 2, along with the small changes in the internal r(AeX) bond length upon forming each complex. The compact F^−^ anion gets closest to Ae, with R(Ae··F) distances less than 3 Å. These intermolecular contacts are slightly longer for CN- and then make a larger jump up to about 3.5 Å for the larger Cl^−^ anion. The larger size of Xe vs. Kr causes a roughly 0.1–0.2 Å elongation, even though V_S,max_ has become slightly less negative. However, the 10 kcal/mol less negative MEP for XeCl_5_^−^ as compared to XeF_5_^−^ allows a slightly closer approach.

The polarity of the solvent also has an effect on the intermolecular distance, but not a simple one. First taking the case of F^−^ approaching KrF_5_^−^, reducing ε first shortens the contact and then expands it, whereas the intermolecular contact shortens with smaller ε for the two XeX_5_^−^ anions. This distance elongates for Cl^−^ approaching XeF_5_^−^, but changes in the opposite direction for XeCl_5_^−^. There is also the observation that Cl^−^ will not engage in a stable complex with KrF_5_^−^ in the low-dielectric THF, whereas both other anions do so.

Also reported in Table 2 are the perturbations of the internal r(Ae-X) bonds within the AeX_5_^−^ units. These changes are fairly small, less than 0.01 Å. The Kr-F bonds are little affected by complexation with an anion. Xe-F bonds are stretched whereas Xe-Cl bonds tend to contract. The nature of the solvent has little effect on these perturbations.

The energetics of the formation of each of these dyads are reported in Table 3. The interaction and binding energies refer respectively to the monomers in their geometries within the complex or within their isolated optimized states. These two quantities thus differ only by the deformation energy required of each monomer to adopt to its structure within the complex. These deformation energies are quite small, as reflected by the near similarity between E_int_ and E_b_. Negative values indicate that the complex is more stable than are the separated monomers.

The complexes in water have the most consistently exothermic energetics, even if the dimerization energies are fairly small, less than 2 kcal/mol. XeCl_5_^−^ forms the most stable complexes, consistent with its least negative V_S,max_, while the competition between KrF_5_^−^ and XeF_5_^−^ depends upon the particular anion. As the polarity of the solvent diminishes, there is a clear trend toward more positive binding energies, such that all quantities are positive for THF. In fact, Cl^−^ will not engage in a complex with KrF_5_^−^ at all in THF.

A partitioning of each interaction energy into its various composite parts leads to the quantities displayed in Table 4. The electrostatic term is consistently repulsive, dropping in the sequence F^−^ > Cl^−^ > NC^−^ and KrF_5_^−^ > XeF_5_^−^ > XeCl_5_^−^ as Lewis acids. This pattern is characteristic of all solvents. The repulsion term disfavors formation of complexes but is dwarfed by the repulsive electrostatic component. In most of the dimers, these two repulsive components are overcome by the three attractive terms: exchange, polarization and dispersion. Among these the most important is polarization which accounts for more than 90% of the sum of all attractive terms. The contributions of dispersion and exchange are comparable, not exceeding more than 5%.

For those systems where the association is endothermic, there is the question as to why the dimer does not simply dissociate. This dissociation is impeded by an energy barrier which the system must surmount. These barriers were estimated by generating the dissociation potential by pulling the two monomers apart in 0.2 Å steps, with no further geometry optimization. Corresponding values are listed in Table 5 for those systems for which the interaction energies of the dimers were positive, which included all complexes in THF and those involving KrF_5_^−^ and XeF_5_^−^ in DMF. The data suggest that these barriers are not very high, no more than 2–3 kcal/mol. The highest of these barriers is associated with the XeCl_5_^−^ unit, regardless of the particular anion. Dissociation barriers occur at various intermolecular distances, varying from less than 4 Å up to distances exceeding 6 Å in DMF.

The QTAIM molecular diagrams of the various complexes in each solvent revealed the presence of a single bond critical point between the central Ae atom and the incoming anion, with no secondary interactions. The Ae··An interaction is clearly noncovalent with a bond critical point density of 0.01–0.02 au, which is only a fraction of the same quantity for the internal Ae-X covalent bonds. This range is consistent with reports of Carvalho and de Araujo Olivera mentioned earlier [52,53]. This density for the Ae··An interaction is catalogued in Table 6, along with its Laplacian as well as the total electron energy density H, for all of the relevant dimers. Comparisons suggest the strongest bond is that with fluoride, which is not fully consistent with the actual energetics in Table 3. This discrepancy is likely due to the much shorter inter-anion distances for F^−^, as the dependence of ρ_BCP_ upon R has been extensively documented. The positive Laplacians are commensurate with its characterization as a noncovalent bond, as is the small positive value of H. There is little sensitivity of any of these AIM properties to the particular solvent.

## 4. Discussion

While it may be notable that a pair of anions can form a complex, even a metastable one, the very weak binding in the aerogen bonds is a point of particular interest. The binding energies do not exceed 2 kcal/mol, even in the strongly polar water solvent. This behavior contrasts with binding energies of various other anion pairs. Taking aqueous solution for the sake of consistency, the binding energy of CN^−^ with the various ACl_3_^−^ anions, where A is a member of Group 2A of the periodic table can be quite a bit larger, ranging all the way up to 20 kcal/mol [82] for A=Be. Similarly large binding energies occur when A is a 2B element Zn, Cd, or Hg [83]. Pnicogen bonds between anions are even larger in magnitude, more than 20 kcal/mol for the ZCl_4_^−^ series, with Z=P, As or Sb [79].

It is perhaps not surprising that the electrostatic component of the interaction energies in these anion-anion complexes is a large positive value, strongly repulsive. It is only because of larger attractive components, chiefly polarization, that these dimers are able to form at all. Here again, these AeBs differ from the other anion-anion complexes discussed above. The electrostatic component is very small for the Group 2A complexes, and its sign depends on the specific central A atom [82]. The electrostatic energy is rather attractive for the Group 2B analogues between 40 and 100 kcal/mol [83] and ramps up to even larger negative amounts even as much as 111 kcal/mol for the pnicogen-bonded anion pairs [79].

**Figure 3:**
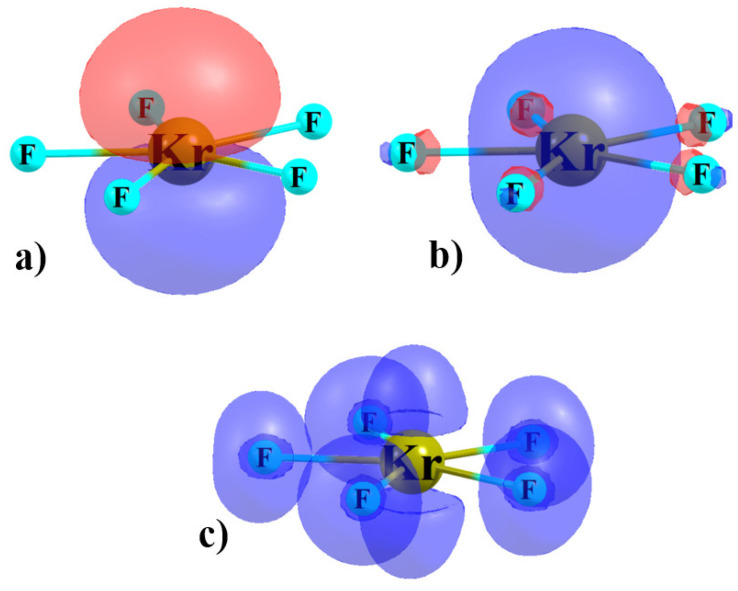
Doubly occupied NBO (**a**) *p* and (**b**) *s*-orbitals of KrF_5_^−^ monomer, and (**c**) ELF diagram.

One may not have anticipated that a π-hole might develop directly above the AeX_5_^−^ anion, albeit one of negative sign. A simple VSEPR analysis of this anion suggests the central Ae atom ought to contain two lone electron pairs. Given the D_5h_ geometry of this unit, these pairs should be disposed directly above and below the central Ae, coinciding with the π-hole. The two NBO lone pair orbitals of KrF_5_^−^ are illustrated in Figure 3a,b. The *p*-orbital of Kr in Figure 3a lies above and below the molecular plane and the *s*-orbital is of course symmetric. Both of these orbitals will contribute electron density to the regions directly above and below the Kr atom. An alternate view combines these two atomic orbitals into a pair of *sp* orbitals, one lying above and one below the molecular plane. This disposition of these two electron pairs is reinforced by the ELF diagram in Figure 3c. Despite the positioning of these two electron pairs, there is indeed a maximum in the MEP that occurs directly along the C_5_ axis which seems capable of attracting the anion. It is not only on the ρ = 0.001 au isosurface that these maxima are so positioned; the same is true of a range of densities. This maximum is likely due to the ability of the five X substituents to draw electron density toward themselves, and out of the region perpendicular to the molecular plane. Nonetheless, the coincidence of the positions of the π-holes and the two Ae lone pairs represents a major factor in the very weak nature of the aerogen bonding in these complexes.

Again drawing a comparison to the other anions mentioned above, the Group 2A and 2B ACl_3_^−^ anions are planar, as is AeX_5_^−^ here [82,83]. However, the central A atom does not have any lone pairs that point directly along their C_3_ axis that would inhibit the approach of an anion from this direction. The central Z pnicogen atom of planar ZCl_4_^−^ contains only a single lone pair [83], whose NBO orbital shape resembles an isotropic *s*-orbital, as in Figure 3b, so is not directed toward the π-hole. It is thus partly for this reason that the binding energies of these various Lewis acid anions with another anion are so much larger than those of the AeBs here.

As noted above, there are a number of complexes which represent metastable minima in the sense that the energy of the complex is higher than that of the separated monomers, and that the dissociation of the complex is impeded by an energy barrier. The data in Table 4 indicate that this barrier is rather shallow, on the order of 2 kcal/mol or less. The idea of a metastable complex between a pair of anions is reminiscent of what has been seen earlier in a number of cases. Previous computations have estimated the dissociation barriers to be considerably higher than those observed here [111]. These barriers are roughly 20 kcal/mol when CN^−^ is added to ACl_3_^−^ where A represents Group IIA atoms Be-Ba [82], and somewhat higher, around 25 kcal/mol, when it is a Group IIB atom Zn, Cd, or Hg that lies at the center [83]. The same magnitude barrier to dissociation occurs for ZCl_4_^−^ anions where Z is a 5A pnicogen atom [79].

## 5. Conclusions

The various AeF_5_^−^ anions are capable of forming an aerogen bonded complex with any of several small anions within the context of a polar medium, but not in vacuo. XeCl_5_^−^ forms the most stable complexes, consistent with its least negative V_S,max_, while the competition between KrF_5_^−^ and XeF_5_^−^ depends upon the particular anion. The anion approaches the central Ae atom from directly above the plane of the D_5h_ species, and the intermolecular distances are slightly shorter than 3 Å. The AeBs are weak, less than 2 kcal/mol relative to the fully separated pair of anions. Even in those cases where the complex is higher in energy than the separate monomers, within the less polar media, there is a low energy barrier impeding the dissociation process.

## Figures and Tables

**Table 1:** Ae-X bond lengths in AeX_5_^−^ monomers and maximum on the ρ = 0.001 au isodensity surface calculated at the MP2/aug-cc-pVDZ level of theory in different solvents.

	r(Ae-X) ^a^, Å	V_S,max_, kcal/mol
	Water (ε = 78.4)	
KrF_5_^−^	2.034	−67.1
XeF_5_^−^	2.066	−61.3
XeCl_5_^−^	2.728	−51.7
	DMF (ε = 37.2)	
KrF_5_^−^	2.034	−67.2
XeF_5_^−^	2.066	−61.4
XeCl_5_^−^	2.729	−51.8
	THF (ε = 7.4)	
KrF_5_^−^	2.036	−67.5
XeF_5_^−^	2.066	−62.0
XeCl_5_^−^	2.730	−52.4

^a^ mean values.

**Table 2:** Intermolecular distances and change in internal bond lengths (Å) upon complex formation.

	An=F^−^		An=Cl^−^		An=CN^−^	
	R(Ae···An)	Δr(Ae-X)	R(Ae···An)	Δr(Ae-X)	R(Ae···An)	Δr(Ae-X)
Water (ε = 78.4)						
KrF_5_^−^	2.848	−0.001	3.406	0.000	3.083	−0.001
XeF_5_^−^	2.981	0.006	3.564	0.003	3.296	0.003
XeCl_5_^−^	2.894	−0.008	3.458	−0.006	3.165	−0.008
DMF (ε = 37.2)						
KrF_5_^−^	2.839	−0.001	3.409	0.000	3.086	−0.001
XeF_5_^−^	2.979	0.006	3.563	0.003	3.296	0.003
XeCl_5_^−^	2.888	−0.007	3.461	−0.007	3.169	−0.008
THF (ε = 7.4)						
KrF_5_^−^	2.860	−0.001	a	a	3.104	−0.001
XeF_5_^−^	2.939	0.008	3.658	0.004	3.304	0.004
XeCl_5_^−^	2.837	−0.008	3.438	0.003	3.170	−0.007

^a^ complex not formed.

**Table 3:** Interaction energy E_int_ and binding energy E_b_ of AeX_5_^−∙^∙∙An^−^ complexes calculated in different solvents at the MP2/aug-cc-pVDZ level of theory. All values in kcal/mol, corrected for BSSE.

	E_int_			E_b_		
	F^−^	Cl^−^	NC^−^	F^−^	Cl^−^	NC^−^
Water (ε = 78.4)						
KrF_5_^−^	−0.56	−0.62	−0.96	−0.50	−0.63	−0.98
XeF_5_^−^	−0.74	−0.43	−0.69	−0.64	−0.41	−0.68
XeCl_5_^−^	−1.51	−1.81	−2.05	−1.49	−1.81	−2.04
DMF (ε = 37.2)						
KrF_5_^−^	0.50	0.33	−0.09	0.58	0.33	−0.09
XeF_5_^−^	0.22	0.52	0.20	0.35	0.56	0.24
XeCl_5_^−^	−0.57	−0.85	−1.14	−0.52	−0.84	−1.13
THF (ε = 7.4)						
KrF_5_^−^	8.36	a	7.04	8.63	a	7.12
XeF_5_^−^	7.30	7.32	6.69	7.71	7.45	6.85
XeCl_5_^−^	6.02	5.80	5.19	6.15	5.87	5.26

a: complex not formed.

**Table 4:** Decomposition of interaction energy (kcal/mol) by LMOEDA scheme calculated at M06-2X/aug-cc-pVDZ level. Dissection terms are as follows: exchange (E_ex_), electrostatic (E_elec_), repulsion (E_rep_), polarization (E_pol_) and dispersion (E_disp_) ^a^.

AeX_5_^−^	R	E_int_	E_ex_	%	E_elec_	E_rep_	E_pol_	%	E_disp_	%
Water (ε = 78.4)										
KrF_5_^−^	F^−^	−6.17	−5.41	2.4	202.99	16.96	−215.18	95.2	−5.53	2.4
	Cl^−^	−3.05	−3.89	1.9	189.54	12.44	−195.79	95.5	−5.35	2.6
	NC^−^	−3.30	−3.87	2.0	182.56	12.40	−189.76	95.7	−4.63	2.3
XeF_5_^−^	F^−^	−5.84	−6.71	3.0	198.54	19.20	−211.35	94.5	−5.52	2.5
	Cl^−^	−2.61	−4.41	2.2	186.30	12.87	−192.43	95.4	−4.94	2.4
	NC^−^	−2.23	−7.32	3.7	177.30	20.20	−186.96	93.6	−5.45	2.7
XeCl_5_^−^	F^−^	−8.26	−8.51	4.0	180.92	24.98	−197.67	92.3	−7.98	3.7
	Cl^−^	−4.64	−6.03	3.1	171.40	18.29	−180.01	92.6	−8.29	4.3
	NC^−^	−4.43	−5.67	3.0	165.20	17.24	−174.31	93.3	−6.89	3.7
DMF (ε = 37.2)										
KrF_5_^−^	F^−^	−5.20	−5.59	2.5	200.76	17.48	−212.25	95.0	−5.60	2.5
	Cl^−^	−2.05	−3.88	1.9	187.67	12.38	−192.92	95.5	−5.30	2.6
	NC^−^	−2.33	−3.85	2.0	180.76	12.34	−186.99	95.7	−4.59	2.3
XeF_5_^−^	F^−^	−4.92	−6.78	3.1	196.39	19.38	−208.38	94.4	−5.53	2.5
	Cl^−^	−1.68	−4.44	2.2	184.37	12.94	−189.62	95.3	−4.93	2.5
	NC^−^	−1.33	−7.34	3.7	175.49	20.24	−184.29	93.5	−5.44	2.8
XeCl_5_^−^	F^−^	−7.45	−8.71	4.1	178.74	25.49	−194.98	92.1	−7.99	3.8
	Cl^−^	−3.75	−6.00	3.1	169.61	18.18	−177.34	92.6	−8.20	4.3
	NC^−^	−3.58	−5.63	3.1	163.54	17.11	−171.78	93.2	−6.82	3.7
THF (ε = 7.4)										
KrF_5_^−^	F^−^	2.51	−5.38	2.7	185.63	16.71	−189.21	94.7	−5.24	2.6
	Cl^−^	not stable								
	NC^−^	4.73	−3.74	2.1	167.44	11.92	−166.57	95.4	−4.32	2.5
XeF_5_^−^	F^−^	1.75	−7.86	3.9	180.17	22.30	−186.94	93.1	−5.93	3.0
	Cl^−^	5.35	−3.52	2.0	170.97	10.40	−168.30	95.6	−4.20	2.4
	NC^−^	5.26	−7.37	4.2	162.15	20.21	−164.45	92.9	−5.28	3.0
XeCl_5_^−^	F^−^	−1.57	−10.50	5.4	162.70	30.18	−175.65	90.3	−8.30	4.3
	Cl^−^	2.65	−6.19	3.6	156.42	18.47	−158.19	91.8	−7.86	4.6
	NC^−^	2.55	−5.87	3.5	151.02	17.65	−153.58	92.5	−6.67	4.0

^a^ also listed as % is the percentage contribution of each component to the total of all attractive terms.

**Table 5:** Estimated barrier to dissociation (kcal/mol) and the intermolecular distance at which it occurs (Å) calculated at MP2/aug-cc-pVDZ level.

	E_diss_	R_diss_	E_diss_	R_diss_	E_diss_	R_diss_
	R=F^−^		R=Cl^−^		R=NC^−^	
DMF (ε = 37.2)						
KrF_5_^−^	1.89	5.24	1.75	6.01	negative E_int_	
XeF_5_^−^	2.00	4.38	1.47	6.16	2.17	6.50
THF (ε = 7.4)						
KrF_5_^−^	0.97	3.66	a		1.25	4.30
XeF_5_^−^	1.43	3.94	0.30	4.66	1.01	4.50
XeCl_5_^−^	2.50	4.04	2.09	4.97	2.73	4.57

a: complex not formed.

**Table 6:** QTAIM descriptors of the AeX_5_^−^∙∙∙R^−^ complexes. Bond critical point (BCP) properties: electron density *ρ*, Laplacian of electron density ∇^2^*ρ* and total electron energy H, were obtained at the MP2/aug-cc-pVDZ level. Data in atomic units.

T_a_	*ρ*	∇ ^2^ *ρ*	H	*ρ*	∇^2^*ρ*	H	*ρ*	∇ ^2^ *ρ*	H
Water (ε = 78.4)									
	An=F^−^			An=Cl^−^			An=NC^−^		
KrF_5_^−^	0.017	0.074	0.002	0.010	0.034	0.002	0.012	0.047	0.002
XeF_5_^−^	0.018	0.070	0.001	0.010	0.033	0.001	0.011	0.039	0.002
XeCl_5_^−^	0.020	0.080	0.001	0.012	0.040	0.001	0.014	0.050	0.002
DMF (ε = 37.2)									
	An=F^−^			An=Cl^−^			An=NC^−^		
KrF_5_^−^	0.017	0.075	0.002	0.010	0.034	0.002	0.012	0.047	0.002
XeF_5_^−^	0.018	0.070	0.001	0.010	0.033	0.001	0.011	0.039	0.002
XeCl_5_^−^	0.020	0.081	0.001	0.012	0.039	0.002	0.014	0.049	0.002
THF (ε = 7.4)									
	An=F^−^			An=Cl^−^			An=NC^−^		
KrF_5_^−^	0.016	0.071	0.002	a			0.012	0.045	0.002
XeF_5_^−^	0.019	0.075	0.001	0.009	0.025	0.001	0.011	0.038	0.001
XeCl_5_^−^	0.023	0.090	0.001	0.013	0.037	0.001	0.014	0.049	0.002

a: complex not formed.

## Data Availability

The data presented in this study are available in this article or Supplementary Material.

## Supplementary Materials

The following are available online. Table S1: Comparison of the total electronic energies (in Hatree) of the Ae···CN^−^ and Ae···NC^−^ complexes.

## Sample Availability

Samples of the compounds are not available from the authors.
