# Supplementary material for: Anion–Anion Interactions in Aerogen-Bonded Complexes. Influence of Solvent Environment
Source: Molecules. 2021 Apr 7;26(8):2116. doi: 10.3390/molecules26082116 (PMC8067769; doi:10.3390/molecules26082116)
Supplement: Supplementary file 1 [file molecules-26-02116-s001.pdf]

Anion-anion interaction in aerogen-bonded complexes, influence of different solvent environments.

Anna Grabarz,[1] Mariusz Michalczyk,[1] Wiktor Zierkiewicz*[1] and Steve Scheiner*[2]

[1] Faculty of Chemistry, Wrocław University of Science and Technology, Wybrzeże Wyspiańskiego 27, 50-370 Wrocław, Poland

[2] Department of Chemistry and Biochemistry, Utah State University Logan, Utah 84322-0300, United States

Table S1 Comparison of the total electronic energies (in Hatree) of the Ae ··CN⁻ and Ae ··NC⁻ complexes.

	NC^-	CN^-	Δ [kcal/mol]
	Water (ε=78.4)		
KrF_5^-	-3342.881852	-3342.881053	0.50
XeF_5^-	-919.3320528	Not formed	
$KrCl_5^-$	Not formed		
$XeCl_5^-$	-2719.387777	-2719.387297	0.30
	DMF (ε=37.2)		
KrF_5^-	-3342.8775042	-3342.8766576	0.53
XeF_5^-	-919.3278021	Not formed	
$KrCl_5^-$	Not formed		
$XeCl_5^-$	-2719.3837527	-2719.3833685	0.24
	THF (ε=7.4)		
KrF_5^-	-3342.8449949	-3342.8442652	0.46
XeF_5^-	-919.2958966	Not formed	
$KrCl_5^-$	Not formed		
$XeCl_5^-$	-2719.3539858	-2719.3535801	0.25

COORDINATES

Monomers

	Geometries		Geometries
Water (ε=78.4)			
KrF_5^-	Kr -0.73009 0.89974 0.00000<br>F -1.92605 -0.74551 0.00000<br>F 0.46588 -0.74551 0.00000<br>F -2.66493 1.52764 0.00000<br>F 1.20476 1.52764 0.00000<br>F -0.73009 2.93420 0.00000	CN^-	C 0.00000 0.00000 0.00000<br>N 1.18900 0.00000 0.00000
XeF_5^-	Xe -0.73009 0.89952 0.00000<br>F 1.23467 1.53788 0.00000<br>F 0.48454 -0.77150 0.00000<br>F -1.94472 -0.77150 0.00000<br>F -2.69485 1.53788 0.00000<br>F -0.73009 2.96597 0.00000		
$KrCl_5^-$	Not formed		
$XeCl_5^-$	Xe -0.73009 0.89943 0.00000<br>Cl 1.86401 1.74325 0.00000<br>Cl 0.87325 -1.30733 0.00000<br>Cl -2.33343 -1.30733 0.00000<br>Cl -3.32419 1.74325 0.00000<br>Cl -0.73009 3.62698 0.00000		
DMF (ε=37.2)			
KrF_5^-	Kr -0.73009 0.89972 0.00000<br>F -1.92619 -0.74574 0.00000<br>F 0.46602 -0.74574 0.00000<br>F -2.66510 1.52781 0.00000<br>F 1.20493 1.52781 0.00000<br>F -0.73009 2.93435 0.00000	CN^-	C -0.11066 0.72710 1.71258<br>N 0.27800 0.62223 2.84726
XeF_5^-	Xe -0.73009 0.89946 0.00000<br>F 1.23464 1.53805 0.00000<br>F 0.48454 -0.77151 0.00000<br>F -1.94472 -0.77151 0.00000<br>F -2.69482 1.53805 0.00000<br>F -0.73009 2.96573 0.00000		
$KrCl_5^-$	Not formed		
$XeCl_5^-$	Xe -0.69729 0.90013 0.18484<br>Cl -0.71416 3.62742 0.09556<br>Cl 1.85924 1.74282 -0.27160<br>Cl 0.89125 -1.30762 -0.03689<br>Cl -2.27067 -1.30836 0.49659<br>Cl -3.25328 1.74317 0.64097		

THF (ε=7.4)			
KrF_5^-	Kr -0.73009 0.89958 0.00000<br>F -1.92705 -0.74747 0.00000<br>F 0.46687 -0.74747 0.00000<br>F -2.66634 1.52908 0.00000<br>F 1.20616 1.52908 0.00000<br>F -0.73009 2.93540 0.00000	CN^-	C -0.11073 0.72712 1.71238<br>N 0.27807 0.62221 2.84746
XeF_5^-	Xe -0.73009 0.89912 0.00000<br>F 1.23444 1.53903 0.00000<br>F 0.48454 -0.77167 0.00000<br>F -1.94472 -0.77167 0.00000<br>F -2.69462 1.53903 0.00000<br>F -0.73009 2.96441 0.00000		
$KrCl_5^-$	Not formed		
$XeCl_5^-$	Xe -0.70461 0.89991 -0.14886<br>Cl -0.65753 3.62541 -0.32860<br>Cl 1.83755 1.73579 0.37613<br>Cl 0.81794 -1.30534 0.38306<br>Cl -2.27246 -1.30711 -0.49498<br>Cl -3.24480 1.74959 -0.67497		

Complexes

	$R=F^-$	$R=Cl^-$	$R=NC^-$
Water (ε=78.4)			
KrF_5^-	Kr -0.68262 0.88625 -0.14346<br>F 1.20835 1.52776 -0.52624<br>F 0.47129 -0.74553 -0.51904<br>F -1.86482 -0.76278 -0.00885<br>F -2.57619 1.50341 0.26463<br>F -0.67440 2.91740 -0.04041<br>F -0.11702 0.70300 2.64224	Kr -0.73251 0.90231 -0.22208<br>F 1.13945 1.54540 -0.68886<br>F 0.40210 -0.72793 -0.66146<br>F -1.90663 -0.74866 -0.04088<br>F -2.59806 1.51377 0.31019<br>F -0.71280 2.93178 -0.07670<br>Cl 0.17303 0.61284 3.04866	Kr -0.66572 0.88192 -0.18985<br>F 1.21345 1.52530 -0.62298<br>F 0.47986 -0.74852 -0.59463<br>F -1.83712 -0.77043 -0.01005<br>F -2.54111 1.49325 0.30227<br>F -0.65455 2.91175 -0.06985<br>N 0.05563 0.69726 2.80234<br>C 0.35583 0.56309 3.96062
XeF_5^-	Xe -0.67270 0.87054 -0.16790<br>F 1.26489 1.52134 -0.50611<br>F 0.50625 -0.79492 -0.53695<br>F -1.89077 -0.80519 -0.09215<br>F -2.60527 1.50121 0.22642<br>F -0.65647 2.93610 -0.02600<br>F -0.18136 0.80043 2.77154	Xe -0.67883 0.87380 -0.25926<br>F 1.26563 1.52321 -0.53662<br>F 0.51104 -0.78991 -0.57694<br>F -1.89364 -0.80082 -0.19813<br>F -2.61828 1.50305 0.08959<br>F -0.66664 2.93678 -0.11474<br>Cl -0.15470 0.78339 3.26497	Xe -0.72513 0.88349 -0.22916<br>F 1.22238 1.52538 -0.49031<br>F 0.46099 -0.78424 -0.52911<br>F -1.94830 -0.78689 -0.18317<br>F -2.66901 1.52157 0.09383<br>F -0.70555 2.94733 -0.07974<br>N 0.22184 0.65065 2.91901<br>C 0.54904 0.59631 4.07654
$KrCl_5^-$	Not formed		

$XeCl_5^-$	Xe -0.65188 0.87652 -0.15381<br>Cl 1.91988 1.72123 -0.41970<br>Cl 0.92994 -1.31944 -0.42259<br>Cl -2.24563 -1.32511 -0.08281<br>Cl -3.22582 1.71205 0.12110<br>Cl -0.65152 3.59504 -0.09160<br>F -0.31040 0.76923 2.71828	Xe -0.70414 0.89202 0.21063<br>Cl 1.88342 1.73393 0.29170<br>Cl 0.89238 -1.30902 0.32912<br>Cl -2.30094 -1.31100 0.14615<br>Cl -3.29130 1.73349 0.12507<br>Cl -0.70281 3.61300 0.17475<br>Cl -0.53451 0.83000 -3.24257	Xe -0.66603 0.86300 -0.22138<br>Cl 1.88711 1.70756 -0.62423<br>Cl 0.89406 -1.33302 -0.60473<br>Cl -2.27275 -1.33131 -0.16345<br>Cl -3.19667 1.68870 0.34143<br>Cl -0.63989 3.57632 -0.02794<br>N 0.03034 0.73775 2.86307<br>C 0.37010 0.64462 4.01511
DMF (ε=37.2)			
KrF_5^-	Kr -0.68133 0.88718 -0.14047<br>F 1.20807 1.52863 -0.52979<br>F 0.47263 -0.74460 -0.51934<br>F -1.86209 -0.76224 -0.00183<br>F -2.57524 1.50402 0.26555<br>F -0.67311 2.91927 -0.04253<br>F -0.12436 0.69725 2.63725	Kr -0.73259 0.90400 -0.22068<br>F 1.13892 1.54763 -0.68891<br>F 0.40373 -0.72635 -0.65582<br>F -1.90510 -0.74733 -0.03594<br>F -2.60048 1.51571 0.30372<br>F -0.71554 2.93444 -0.08467<br>Cl 0.17564 0.60142 3.05117	Kr -0.66226 0.88126 -0.18999<br>F 1.21293 1.52516 -0.63984<br>F 0.48007 -0.74868 -0.60797<br>F -1.83184 -0.77179 -0.00534<br>F -2.53456 1.49124 0.31524<br>F -0.65051 2.91128 -0.06800<br>N 0.04964 0.70298 2.80719<br>C 0.34279 0.56215 3.96658
XeF_5^-	Xe -0.67075 0.86879 -0.16598<br>F 1.26635 1.51965 -0.50824<br>F 0.50789 -0.79639 -0.53970<br>F -1.89011 -0.80666 -0.09804<br>F -2.60330 1.49885 0.22919<br>F -0.65489 2.93395 -0.02168<br>F -0.19062 0.81133 2.77332	Xe -0.67618 0.87540 -0.25772<br>F 1.26597 1.52567 -0.55071<br>F 0.51314 -0.78885 -0.57663<br>F -1.89012 -0.79957 -0.18875<br>F -2.61483 1.50442 0.09511<br>F -0.66524 2.93888 -0.12019<br>Cl -0.16816 0.77357 3.26775	Xe -0.72556 0.88233 -0.22784<br>F 1.22237 1.52401 -0.48428<br>F 0.45897 -0.78549 -0.53242<br>F -1.95102 -0.78694 -0.19185<br>F -2.67030 1.52097 0.09089<br>F -0.70561 2.94589 -0.07347<br>N 0.22541 0.64563 2.91924<br>C 0.55200 0.60720 4.07760
$KrCl_5^-$	Not formed		
$XeCl_5^-$	Xe -0.65490 0.87397 -0.14861<br>Cl 1.91583 1.71874 -0.42523<br>Cl 0.92595 -1.32177 -0.42279<br>Cl -2.24908 -1.32793 -0.07709<br>Cl -3.23218 1.71093 0.08716<br>Cl -0.65249 3.59186 -0.05962<br>F -0.28856 0.78370 2.71503	Xe -0.70757 0.88956 0.20721<br>Cl 1.88040 1.73176 0.27348<br>Cl 0.88811 -1.31154 0.33539<br>Cl -2.30628 -1.31276 0.15764<br>Cl -3.29530 1.73103 0.13501<br>Cl -0.70715 3.61093 0.17428<br>Cl -0.51010 0.84345 -3.24816	Xe -0.67215 0.86015 -0.21749<br>Cl 1.88319 1.70240 -0.61237<br>Cl 0.88860 -1.33847 -0.58428<br>Cl -2.28600 -1.33001 -0.19785<br>Cl -3.20476 1.68733 0.33558<br>Cl -0.64608 3.57357 -0.02733<br>N 0.06715 0.76662 2.86249<br>C 0.37632 0.63201 4.01911
THF (ε=7.4)			
KrF_5^-	Kr -0.66678 0.88508 -0.13407<br>F 1.21060 1.52765 -0.58106<br>F 0.47149 -0.74582 -0.57770<br>F -1.84118 -0.76830 0.03246<br>F -2.56261 1.49807 0.26543<br>F -0.65584 2.91906 -0.01950<br>F -0.19109 0.71378 2.68330	Not formed	Kr -0.64587 0.88761 -0.18894<br>F 1.20498 1.53498 -0.73946<br>F 0.48236 -0.74404 -0.64925<br>F -1.81468 -0.76658 0.00252<br>F -2.50360 1.49379 0.37749<br>F -0.63128 2.91876 -0.05920<br>N 0.00941 0.69437 2.83894<br>C 0.30494 0.53471 3.99576
XeF_5^-	Xe -0.70553 0.88978 -0.13650<br>F 1.24182 1.53764 -0.38851	Xe -0.67434 0.91500 -0.25668<br>F 1.22907 1.53576 -0.79752	Xe -0.72694 0.89290 -0.22049

	F 0.48899 -0.78064 -0.38593<br>F -1.93017 -0.78709 -0.09771<br>F -2.67471 1.53089 0.05553<br>F -0.70632 2.96283 -0.07327<br>F 0.05051 0.67612 2.69526	F 0.44482 -0.76626 -0.71148<br>F -1.89653 -0.73901 -0.06315<br>F -2.54553 1.57392 0.32345<br>F -0.61980 2.98233 -0.15517<br>Cl -0.17312 0.55779 3.34943	F 1.21458 1.54085 -0.49596<br>F 0.46350 -0.77574 -0.49214<br>F -1.94593 -0.78106 -0.14251<br>F -2.68331 1.53136 0.03891<br>F -0.72237 2.96086 -0.12148<br>N 0.26124 0.65015 2.92335<br>C 0.54549 0.53427 4.08819
$KrCl_5^-$	Not formed		
$XeCl_5^-$	Xe -0.65757 0.87314 -0.12184<br>Cl 1.91362 1.71741 -0.42170<br>Cl 0.92297 -1.32292 -0.40711<br>Cl -2.25633 -1.32964 -0.08947<br>Cl -3.24055 1.71052 0.07456<br>Cl -0.65639 3.59352 -0.05137<br>F -0.26118 0.78749 2.68580	Xe -0.68272 0.87022 0.16691<br>Cl 1.90175 1.72418 0.42422<br>Cl 0.90707 -1.34374 0.36749<br>Cl -2.28250 -1.34359 0.16513<br>Cl -3.27816 1.71859 0.14371<br>Cl -0.68383 3.60114 0.03755<br>Cl -0.63951 0.95563 -3.27018	Xe -0.71115 0.85663 -0.18707<br>Cl 1.85628 1.68906 -0.53571<br>Cl 0.85323 -1.34690 -0.51108<br>Cl -2.33296 -1.32865 -0.24856<br>Cl -3.28292 1.70839 0.11837<br>Cl -0.66955 3.56297 0.08163<br>N 0.15551 0.71389 2.85881<br>C 0.53782 0.69821 4.00149
